# Exploring the link between Thought Disorders and Suicide Risk: evidence from a clinical cohort of adolescents

**DOI:** 10.1192/j.eurpsy.2026.10519

**Published:** 2026-07-31

**Authors:** F. Marazzi, M. Orlandi, R. Borgatti, P. Fusar-Poli, M. M. Mensi

**Affiliations:** 1 Department of Brain and Behavioral Sciences, University of Pavia; 2 Child Neurology and Psychiatry Unit, IRCCS Mondino Foundation, Pavia, Italy; 3 EPIC Lab, Department of Psychosis Studies, Institute of Psychiatry, Psychology and Neuroscience, King’s College London, London, United Kingdom; 4 Department of Psychiatry and Psychotherapy, Ludwig-Maximilian-University of Munich, Munich, Germany

## Abstract

**Introduction:**

Thought disorders in adolescence are associated with significant distress, functional impairment, and increased vulnerability to suicide risk. Similarly, suicide risk is a major public health concern among adolescents. Although evidence suggests an association between suicidal risk and thought disorders in adolescents, the literature on this topic remains limited.

**Objectives:**

This study aimed to characterize the presence and features of thought disorders in a clinical cohort of adolescents, stratified by suicidal risk. We sought to identify differences in vulnerability across different clinical populations (no suicidal concern, low suicidal risk, high suicidal risk) to better understand patterns of susceptibility.

**Methods:**

112 adolescents (12–18 years), admitted to a Child and Adolescent Neuropsychiatry unit (2020-2024), were recruited and underwent comprehensive psychodiagnostic assessments. The Columbia Suicide Severity Rating Scale (C-SSRS) was used to classify patients into no suicidal concern (NSC) and suicidal concern (SC; divideded into low vs high suicide risk), while the Comprehensive Assessment of At-Risk Mental States (CAARMS) assessed the presence and characteristics of thought disorders. Sociodemographic data, social functioning (SOFAS), and clinical severity (CGI-S) were also collected.

**Results:**

The sample was predominantly female (83%), with a mean age of 15.5. Thought disorders were more frequent and severe in patients with SC compared to NSC. Among patients with psychosis or attenuated psychosis, SC was significantly associated with greater severity of formal thought disorders. Even among those without formal thought disorders, SC corresponded to a higher incidence of mild disorganization phenomena. Across all subgroups, SC correlated with poorer social functioning (SOFAS) and greater clinical severity (CGI-S).

**Conclusions:**

Suicidal risk modulates the expression and severity of thought disorders in adolescents, with distinct patterns depending on the clinical profile. Formal thought alterations predominate in psychotic patients, whereas pathological ideational content is more evident in at-risk or non-psychotic subjects. These findings emphasize the importance of evaluating thought disorders not only as markers of psychotic vulnerability but also as critical indicators for assessing suicidal risk. Detailed characterization of ideational processes may support targeted prevention and intervention strategies in the adolescent population.

**Disclosure of Interest:**

None Declared

